# Local and Systemic Immune Mechanisms Underlying the Anti-Colitis Effects of the Dairy Bacterium *Lactobacillus delbrueckii*

**DOI:** 10.1371/journal.pone.0085923

**Published:** 2014-01-21

**Authors:** Clarissa Santos Rocha, Ana Cristina Gomes-Santos, Thais Garcias Moreira, Marcela de Azevedo, Tessalia Diniz Luerce, Mahendra Mariadassou, Ana Paula Longaray Delamare, Philippe Langella, Emmanuelle Maguin, Vasco Azevedo, Ana Maria Caetano de Faria, Anderson Miyoshi, Maarten van de Guchte

**Affiliations:** 1 Department of General Biology, Instituto de Ciências Biológicas, Universidade Federal de Minas Gerais, Belo Horizonte, Brazil; 2 Department of Biochemistry and Immunology, Instituto de Ciências Biológicas, Universidade Federal de Minas Gerais, Belo Horizonte, Brazil; 3 INRA, UMR1319 Micalis, Jouy-en-Josas, France; 4 AgroParisTech, UMR Micalis, Jouy-en-Josas, France; 5 INRA, Mathématique, Informatique et Génome, UR1077, Jouy-en-Josas, France; 6 Institute of Biotechnology, Universidade de Caxias do Sul, Caxias do Sul, Brazil; Charité, Campus Benjamin Franklin, Germany

## Abstract

Several probiotic bacteria have been proposed for treatment or prevention of inflammatory bowel diseases (IBD), showing a protective effect in animal models of experimental colitis and for some of them also in human clinical trials. While most of these probiotic bacteria are isolated from the digestive tract, we recently reported that a *Lactobacillus* strain isolated from cheese, *L. delbrueckii* subsp. *lactis* CNRZ327 (Lb CNRZ327), also possesses anti-inflammatory effects *in vitro* and *in vivo*, demonstrating that common dairy bacteria may be useful in the treatment or prevention of IBD. Here, we studied the mechanisms underlying the protective effects of Lb CNRZ327 *in vivo*, in a mouse dextran sodium sulfate (DSS) colitis model. During colitis, Lb CNRZ327 modulated the production of TGF-β, IL-6, and IL-12 in colonic tissue and of TGF-β and IL-6 in the spleen, and caused an expansion of CD4+Foxp3+ regulatory T cells in the cecal lymph nodes. Moreover, a strong tendency to CD4+Foxp3+ expansion was also observed in the spleen. The results of this study for the first time show that orally administered dairy lactobacilli can not only modulate mucosal but also systemic immune responses and constitute an effective treatment of IBD.

## Introduction

The gastro intestinal tract (GIT) constitutes the largest surface of contact between the body and the external environment [1], and harbors a diverse population of commensal microorganisms that play an important role in the development and functioning of the immune system [2], [3]. Under physiological conditions, the constant exposure to antigens through the gut mucosa leads to local and systemic immunological activities, such as secretory immunoglobulin A (sIgA) production and oral tolerance induction [3], [4].

Inflammatory bowel diseases (IBDs), including ulcerative colitis (UC) and Crohn's disease (CD), are characterized by a spontaneous and chronic inflammation of the GIT. Although the causes for IBD remain obscure, there is growing evidence that IBD results from abnormal immune responses to the gut microbiota in individuals with genetic predisposition. The state of tolerance to the native microbiota is disturbed by the presence of a deregulated effector T cell population that reacts to the normal microbiota or, alternatively, by the presence of a defective Treg (regulatory T-cell) population that does not properly modulate the effector T cell responses [5], [6].

Patients with IBD present an abnormal luminal microbiota and it has been suggested that the lack of bacteria with anti-inflammatory properties observed in the dysbiosis accompanying IBD could be a key factor in the persistence of inflammation [7], [8]. In this context, the administration of probiotics may balance the indigenous microbiota and several have been proposed for IBD treatment, showing a protective effect in animal models of experimental colitis and for some of them also in human clinical trials [9], [10]. Although the mechanisms underlying the protective effects of probiotics in IBD remain largely unknown, some of them have been partially characterized. These include the enhancement of the intestinal barrier function [11], the secretion of antimicrobial compounds [12], and the modulation of intestinal epithelial and mucosal immune cell responses [13]. Several probiotics have been reported to exert anti-inflammatory effects by the modulation of innate and adaptive immune responses. It has been shown that they can modulate the balance between Th1, Th2, Th17 and Treg cells, down-regulate the production of pro-inflammatory cytokines and stimulate anti-inflammatory cytokine production [14], [15].

We recently reported that a dairy *Lactobacillus* isolated from cheese, *Lactobacillus delbrueckii* subsp. *lactis* CNRZ327 (Lb CNRZ327) inhibits TNF-α-induced NF-κB activation in intestinal epithelial cells, *in vitro*, by reducing IκB phosphorylation [16]. The nuclear factor κB is a key transcription factor in the establishment of an inflammatory response and its inhibition has been proposed as an important therapeutic target for IBD [17]. The same *Lactobacillus* strain also attenuated the symptoms of DSS induced colitis in mice [16]. Here, we examine the immune modulation mechanisms underlying its protective effect *in vivo*.

## Materials and Methods

### Ethics statement

Conventional female C57BL/6 mice (Federal University of Minas Gerais, Brazil), 10 to 12 weeks of age, were kept in an environmentally controlled room (21°C, 55% RH) with a 12-h light-dark cycle in the conventional experimental animal facility of Laboratório de Imunobiologia, Instituto de Ciências Biológicas, Universidade Federal de Minas Gerais, Belo Horizonte, Brasil. Mouse chow and water were administered *ad libidum*. At the end of the experiments, mice were sacrificed by cervical dislocation. This study was approved by the local Ethical Committee for Animal Experimentation (CETEA - UFMG, CETEA # 114/2010).

### Bacterial strain

*Lactobacillus delbrueckii* subsp. *lactis* CNRZ327 (Lb CNRZ327) from the INRA collection was grown at 42°C under microaerobic conditions in MRS broth (Difco) or on the same medium solidified with 2% agar.

### DSS-induced colitis

Experiments were performed several times with 5 to 10 mice per group in each experiment (see Figure legends for details on the number of repetitions for each parameter measured). After acclimatization, colonic inflammation was induced by the administration of a 2% (w/v) DSS (Dextran Sodium Sulphate; 40 kDa, ICN n° 160110) aqueous solution as the only source of drinking water for 7 days. All mice were sacrificed at day 8. Liquid consumption was monitored and all mice groups consumed similar volumes of DSS solution daily. Lb CNRZ327 was administered intragastrically from the day before the beginning of the DSS treatment, and daily until day 7. For this purpose, daily cultures of Lb CNRZ327 (OD_600_ = 1) were prepared in MRS medium (Difco). Cultures were centrifuged and collected cells were washed with 0.9% NaCl and resuspended in phosphate buffered saline (PBS; NaCl 8 g/L, KCl 0.2 g/L, NaH_2_PO_4_ 1.44 g/L, K_2_HPO_4_ 0.24 g/L, pH 7.4) to obtain a final concentration of 2.5×10^10^ colony forming units (CFU) per mL. Each mouse received 0.2 mL of bacterial suspension by intragastric administration (5×10^9^ CFU/mouse/day). Negative control mice, called PBS group, received PBS intragastrically, and no DSS was added to their drinking water. Positive control mice (DSS group) received PBS intragastrically, and DSS was added to their drinking water. Animals from the experimental group received Lb CNRZ327 intragastically and DSS was added to their drinking water (327+DSS group).

### Macroscopic analysis

Body weight was assessed at the start of the experiment and daily until the end of the experiment (day 8). Weight change is expressed as percentage change in weight compared with the starting weight. The disease activity index (DAI) was obtained as described by Murthy *et al.* [18] by scoring three major clinical signs: weight loss, diarrhea, and rectal bleeding.

### Histological analysis

The colon was dissected free of fat and mesentery tissue, opened and cleaned with PBS. For histological inflammation scoring, sections of paraffin embedded descendent colon tissue were stained with haematoxylin and eosin, and observed under the microscope. Histological scoring was based on a semiquantitative scoring system described by McCafferty *et al.* [19] in which the following features were graded: extent of destruction of normal mucosal architecture (0, normal; 1, 2, and 3, mild, moderate, and extensive damage, respectively), presence and degree of cellular infiltration (0, normal; 1, 2, and 3, mild, moderate, and transmural infiltration, respectively), extent of muscle thickening (0, normal; 1, 2, and 3, mild, moderate, and extensive thickening, respectively), presence or absence of crypt abscesses (0, absent or 1, present), and presence or absence of goblet cell depletion (0, absent or 1, present). Mice were scored blindly by an expert.

### Measurement of colon and spleen cytokines

Colon or spleen samples were weighed and homogenized in PBS containing 0.05% Tween-20, 0.1 mM phenylmethylsulphonyl fluoride, 0.1 mM benzethonium chloride, 10 mM EDTA and 20 KIU Aprotinin A using a tissue homogenizer (100 mg tissue/ml buffer). Suspensions were centrifuged at 12.000× g for 20 min at 4°C and the supernatants were transferred to microtubes and stored at −80°C until analysis. Concentrations of IL-4, IL-6, IL-10, IL-12, IL-17 and TGF-β were measured by enzyme-linked immunosorbent assay (ELISA) as described by Maron *et al.* [20]. Briefly, supernatants were added to microtitre plates (Nunc), previously coated with purified monoclonal antibodies reactive to IL-4, IL-6, IL-10, IL-12, IL-17 and TGF-β (BD Bioscience). Standards and samples were added and incubated overnight at 4°C. Biotinylated monoclonal antibodies against cytokines were added and incubated for 1 h at room temperature, after which peroxidase-labelled streptavidin (Sigma) was added. A color reaction was developed at room temperature with 100 uL/well of orthophenylenediamine (1 mg/ml) and 0.04% H_2_O_2_ substrate in sodium citrate buffer. The reaction was terminated by the addition of 20 µL/well of 2 N H_2_SO_4_. Absorbance was measured at 492 nm by an ELISA reader (Bio-Rad Model 450 Microplate Reader). Results are presented as a percentage of the values obtained for the negative control group which received the value of 100.

### Measurement of secreted IgA (sIgA)

Fecal samples were weighed and homogenized in PBS (100 mg feces/ml buffer). The suspensions were centrifuged at 12.000 g for 20 min at 4°C, and the levels of sIgA in the supernatant were determined by ELISA as previously described [20].

### Analysis of cell subsets by flow cytometry

Briefly, 1×10^6^ cells isolated from spleen or cecal lymph nodes were resuspended in PBS containing 0.2% BSA (Bovine Serum Albumin) and 0.1% NaN_3_ (PBS-BSA-NaN_3_, pH 7.4). For surface antigen detection, the cells were labeled with monoclonal antibodies (FITC-conjugated anti-mouse CD4 and Cy5-conjugated anti-mouse CD69) for 30 min at 4°C. For intracellular staining, cells were fixed and permeabilized with Fixation/Permeabilization working solution (eBioscience) for one hour on ice and subsequently incubated with PE-conjugated anti-mouse Foxp3 for 30 min at 4°C. The cells were then washed in PBS-BSA-NaN_3_ and centrifuged at 1200× g for 2 min at 4°C. The supernatant was discarded and the cells resuspended in 200 µL of the same buffer containing 1% paraformaldehyde. Cells were acquired using a FACSCan (BECTON & DICKINSON) and data were analyzed by FlowJo (TREESTAR). At least 30,000 events were counted for each sample. Results are presented as a percentage of the negative control group which received the value of 100.

### Statistical analysis

Data were analyzed in accordance with an unbalanced factorial experimental design, as all parameters (e.g. cytokine-levels) were measured in all combinations of tissue and treatment but with unequal sample sizes and not always during the same experiment. The effect of a treatment was examined independently for each parameter within each tissue, when applicable. This led to a total of 20 analyses (2 tissues×6 cytokine levels, 2 tissues×2 cell counts, and weight, DAI, IgA and histological score not related to tissue). Cytokine levels and cell counts showed considerable scale variations between experiments, and were therefore expressed as a percentage of the average level or count in the negative control (PBS) in the same experiment. This type of normalization was not necessary for the parameters weight, DAI, IgA and histological score. For all parameters, a clear (additive) experiment effect was observed which was modeled as a random effect in downstream analyses. To assess the effect of treatment on each parameter, linear mixed models were used as implemented in the lmer function of the R lme4 package [21]. Treatment was used as a fixed effect, Experiment as a random effect, and an interaction [Treatment×Experiment] was added to the model when found significant (p<0.05). The overall effect of the treatment was then assessed with a likelihood ratio test. In case of a significant difference, a post-hoc analysis using Tukey's HSD test was performed to detect significantly different pairs of treatments. All data are expressed as mean ± standard error of the mean (SEM), which are computed under the abovementioned linear mixed model framework. They are the analogs of standard mean and SEM, taking the grouping of mice in independent experiments into account.

The possible existence of correlations between cytokine levels and cell counts in different tissues was analyzed where the experimental data allowed so. Because of the incomplete design, only 16 such correlations (2 tissues×2 cell counts×4 cytokine levels) out of the 24 theoretical ones could be computed. Although the factor “Experiment” still had the potential to be a confounding factor in this analysis, visual inspection confirmed by linear mixed modeling did not show any Experiment effect. Therefore, only simple correlations were computed.

## Results

### *L. delbrueckii* shows anti-inflammatory properties in DSS-induced colitis in C57BL/6 mice

**Figure 1:**
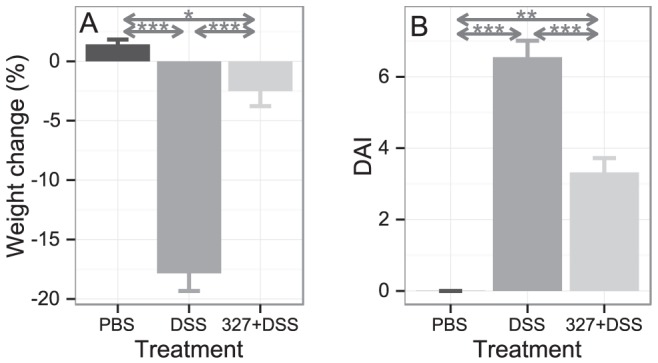
Effect of Lb CNRZ327 on body weight and DAI in DSS-induced colitis. **A**. Body weight change from day 1 to day 8. Each bar represents the average body weight change ± SEM of the 5 mice in the group (one experiment, see text for further details) as a percentage of their initial weights. **B**. Disease activity index (DAI) on day 8. Bars represent the mean ± SEM calculated across three independent experiments, with 5 to 10 mice per group in each experiment (combined sample sizes: 17 to 20 mice per treatment). * = p<0.05; ** = p<0.01; *** = p<0.001.

**Figure 2:**
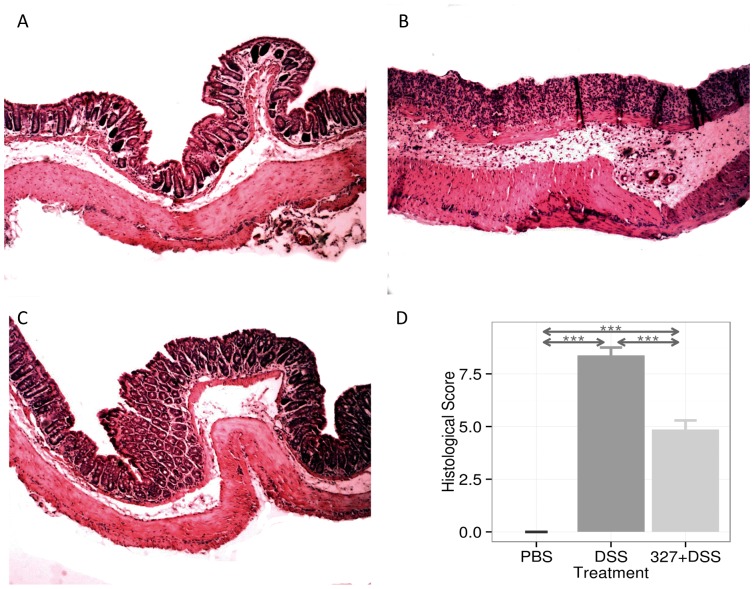
Colon histology after DSS-induced colitis. **A**. Descending colon of the negative control group (without bacteria or DSS). **B**. Descending colon of the positive group control (only DSS). **C**. Descending colon of the experimental group (DSS plus Lb CNRZ327). A, B, and C each show representative results from three independent experiments. **D**. Histological score of the groups described above. Bars represent the mean ± SEM calculated across three independent experiments, with 4 or 5 mice per group in each experiment (combined sample sizes: 13 to 14 mice per treatment). *** = p<0.001.

DSS administration in the drinking water induces an acute inflammation in the gut with symptoms resembling those of human UC [22]. The impact of Lb CNRZ327 on DSS-induced colitis in mice was evaluated through the analysis of the major clinical signs (weight loss, diarrhea, and rectal bleeding), yielding a combined DAI (disease activity index) score, and colon histology, in comparison with mice that received no bacteria (DSS group). All parameters were analyzed at day 8, after the last administration of DSS in the drinking water. In three out of 4 experiments, no difference was observed in weight change over the course of the experiment between the DSS and PBS group, despite clear signs of inflammation (diarrhea and rectal bleeding) in the former (data not shown). In the fourth experiment, PBS-treated mice gained weight (∼1.6%) by the end of the experiment while DSS exposure severely affected the weight of the mice, resulting in a weight loss of about 19%. Administration of Lb CNRZ327 significantly reduced weight loss to about 3% at the end of this experiment (Fig. 1A). Figure 1B shows the DAI at the end of the experiment. The DSS-treated mice without bacteria obtained a score of 6.5 whereas a significantly reduced score of 3.4 was observed in Lb CNRZ327-treated mice. Microscopic examination showed that control mice without DSS presented a normal colon histology (Fig. 2A) while DSS exposure provoked the loss of mucosal architecture with ulcerations, transmural immune cell infiltration through the mucosa and submucosa, edema, goblet cell depletion and increased thickness of the intestinal mucosa and submucosa (Fig. 2B). The administration of Lb CNRZ327 almost abrogated these intestinal changes (Fig. 2C). Colonic tissue damage was given a score taking into account the extent of destruction of the normal mucosal architecture, the presence and degree of cellular infiltration, the extent of muscle thickening, the presence or absence of crypt abscesses and the presence or absence of goblet cell depletion. The DSS-treated group without bacteria obtained a score of 8.9 which was significantly reduced to 5 in Lb CNRZ327-treated mice (Fig. 2D).

### *L. delbrueckii* tends to increase the level of secretory IgA (sIgA)

**Figure 3:**
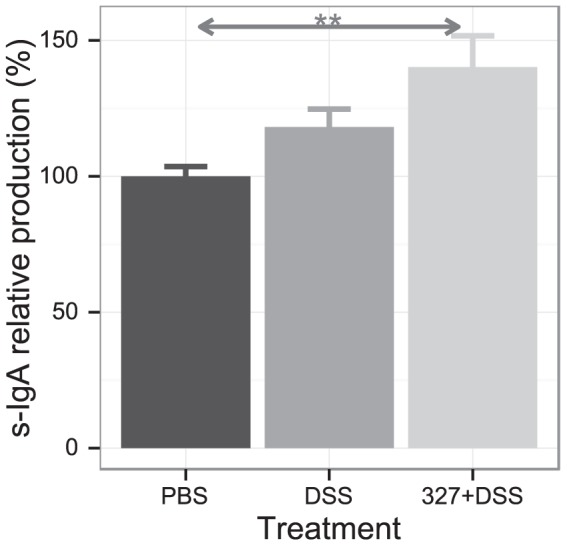
Secretory IgA levels after DSS-induced colitis. Feces were collected and total sIgA was measured by ELISA. PBS, control without bacteria or DSS; DSS, DSS only, without bacteria; 327+DSS, DSS plus Lb CNRZ327. 100 (the average amount observed in the PBS control group) corresponds to the range of 61.5 to 126 pg/mL. Bars represent the mean ± SEM calculated across three independent experiments, with 3 to 10 mice per group in each experiment (combined sample sizes: 15 to 24 mice per treatment). ** = p<0.01.

An increasing number of probiotic strains have been shown to increase IgA secretion, which is important to selectively exclude the entrance of pathogenic agents [23], [24]. We therefore analyzed the impact of Lb. CNRZ327 on the sIgA levels in the feces of DSS-treated mice. As shown in Fig. 3, the level of IgA in DSS-exposed mice did not significantly differ from the levels found in the PBS group. The administration of DSS and Lb CNRZ327 increased the s-IgA level to a level that was significantly higher than in the PBS group, although the difference with the DSS group was not significant.

### *L. delbrueckii* modulates the production of cytokines and T-cell differentiation in colonic tissue

**Figure 4:**
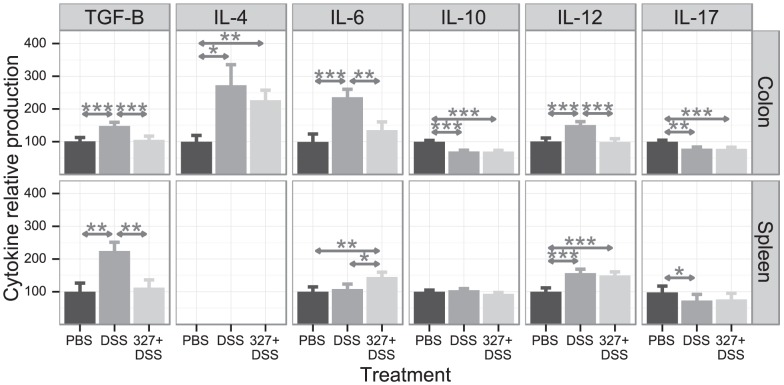
Cytokine production in colonic tissue and spleen after DSS-induced colitis. Cytokine concentrations in homogenized tissues are presented as a percentage of the amount observed in the PBS control group (range from different experiments given in parentheses for each cytokine) which received the value of 100. Upper panel, colon: TGF-β (100 = 69 to 131.4 pg/mL), IL-4 (100 = 70.8 to 161.2 pg/mL), IL-6 (100 = 63.2 to 159.2 pg/mL), IL-10 (100 = 72.8 to 146.2 pg/mL), IL-12 (100 = 71.4 to 168.8 pg/mL), IL-17 (100 = 83.3 to 143.4 pg/mL). Lower panel, spleen: TGF-β (100 = 80.7 to 169.7 pg/mL), IL-6 (100 = 80.1 to 134 pg/mL), IL-10 (100 = 73.1 to 143.2 pg/mL), IL-12 (100 = 53.1 to 123 pg/mL), IL-17 (100 = 68.3 to 136.2 pg/mL). PBS, control without bacteria or DSS; DSS, DSS only, without bacteria; 327+DSS, DSS plus Lb CNRZ327.Bars represent the mean ± SEM calculated across two to six independent experiments, with 2 to 10 mice per group in each experiment (combined sample sizes: 9 to 33 mice per treatment (colon) and 5 to 12 mice per treatment (spleen)). * = p<0.05; ** = p<0.01; *** = p<0.001.

In order to determine if Lb CNRZ327 can modulate DSS-induced cytokines, we measured the levels of TGF-β, IL-4, IL-6, IL-10, IL-12, and IL-17 in colonic tissue. The exposure of C57BL/6 mice to 2% DSS led to significant 1.5, 2.7, 2.4, and 1.5-fold increases in colonic TGF-β, IL-4, IL-6, and IL-12 levels, respectively. The administration of Lb CNRZ327 diminished the production of TGF-β, IL-6 and IL-12 to levels that are not significantly different from those measured in the PBS control group, while the amounts of IL-4 were not significantly affected (Fig. 4, upper panel). Lb CNRZ327 did not affect the decrease in colonic levels of IL-10 and IL-17 induced by the administration of DSS (Fig. 4).

**Figure 5:**
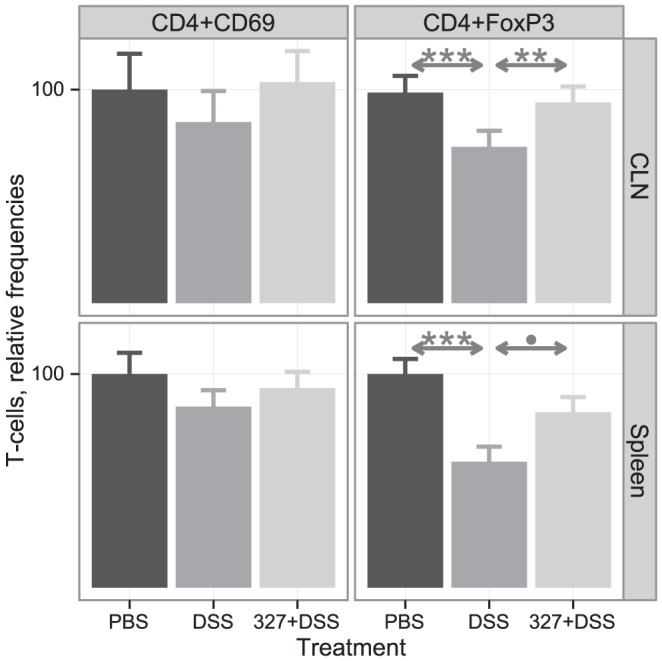
T lymphocyte profiles in cecal lymph nodes and spleen after DSS-induced colitis. T cells were isolated from cecal lymph nodes (CLN, upper panel) and spleen (lower panel) at the end of the experiment, and the frequencies of CD4^+^CD69^+^ and CD4^+^Foxp3^+^ T cells, as percentage of CD4+ T cells, were assessed by flow cytometry. T-cell frequencies are presented relative to the average frequency observed in the PBS control group (range from different experiments given in parentheses for each T cell type) which received the value of 100. Upper panel, CLN: CD4+CD69+ (100 = 2.7 to 3.54), CD4+FoxP3+ (100 = 3.41 to 5.41). Lower panel, spleen: CD4+CD69+ (100 = 4.5 to 4.51), CD4+FoxP3+ (100 = 1.59 to 4.02). PBS, control without bacteria or DSS; DSS, DSS only, without bacteria; 327+DSS, DSS plus Lb CNRZ327. For CD4^+^CD69^+^ T cells, bars represent the mean ± SEM of one experiment, with 2 or 3 mice in the PBS control groups and 5 mice in each of the other groups. For CD4^+^FoxP3+ T cells, bars represent the mean ± SEM calculated across two independent experiments, with 3 to 5 mice per group in each experiment (combined sample sizes: 8 to 10 mice per treatment (colon) and 8 mice per treatment (spleen)). ** = p<0.01; *** = p<0.001; • = p = 0.053.

As patients with IBD present a disrupted balance between effector and regulatory T cells [5], [6], we analyzed the effect of DSS and Lb CNRZ327 administration on the frequencies of T-cell subtypes in cecal lymph nodes (CLN). Neither DSS nor the combination of DSS and Lb CNRZ327 significantly affected the frequency of T cells expressing the early activation marker CD69 in CLN (Fig. 5, upper panel). While T cells with a regulatory phenotype (CD4^+^ Foxp3^+^) were less frequent after DSS treatment (Fig. 5), Lb CNRZ327 restored the levels of Foxp3^+^ cells within the CD4^+^ compartment in the CLN of DSS-treated mice to those found in the control group (Fig. 5).

### Systemic effects of *L. delbrueckii*

In order to see if the effects of the oral administration of *L. delbrueckii* were restricted to the gut and associated tissues or more widespread, we measured TGF-β, IL-6, IL-10, IL-12, and IL-17 in the spleen of the mice. DSS exposure increased the levels of splenic TGF-β and IL-12 (Fig. 4, lower pannel). Lb CNRZ327 abolished the increase in TGF-β, but did not affect IL-12 levels. The secretion of splenic IL-17 was reduced by DSS, and Lb CNRZ327-treatment did not restore the production of this cytokine (Fig. 4). The production of splenic IL-6 was not affected by DSS, but slightly enhanced in the presence of DSS and Lb CNRZ327, and IL-10 was not affected by either DSS or DSS and Lb CNRZ327 (Fig. 4).

**Figure 6:**
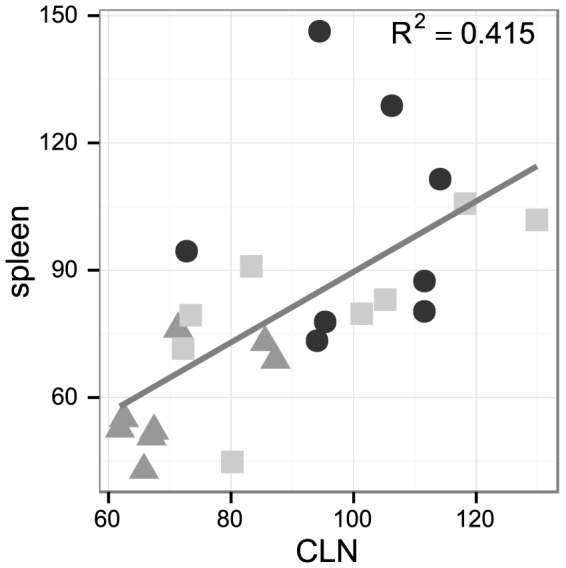
Correlation between relative frequencies of CD4+FoxP3 T cells in CLN and spleen. Each point represents one mouse, placed as a function of relative CD4+FoxP3 T cell frequencies in CLN and spleen (cf legend to Fig. 5). Black dots, PBS (control without bacteria or DSS); grey triangles, DSS (DSS only, without bacteria); light grey squares, 327+DSS (DSS plus Lb CNRZ327). The grey line describes the linear relationship between the frequencies of CD4^+^Foxp3^+^ cells in the spleen and in the colon (R^2^ = 0.415).

Regarding T-cell differentiation, neither DSS nor the combination of DSS and Lb CNRZ327 significantly affected the frequency of T cells expressing the early activation marker CD69 in the spleen (Fig. 5, lower panel). However, while DSS-treatment resulted in an important reduction in the frequency of T cells with a regulatory phenotype (CD4^+^Foxp3^+^) in the spleen (Fig. 5), we observed a strong tendency of augmentation of this cell-type after treatment with Lb CNRZ327 (Fig. 5). This tendency reflects the changes observed in the colon, and is further supported by the strong correlation that can be observed between the frequencies of CD4^+^Foxp3^+^ cells in the spleen and in the colon (Fig. 6).

Together, these results clearly demonstrate that the immune-modulation effects of *L. delbrueckii* go beyond a local effect in gut-associated tissues.

## Discussion

The aim of this study was to characterize the mechanisms involved in the anti-inflammatory effects of Lb CNRZ327 in DSS-induced colitis in mice. We induced colitis in C56BL/6 mice by adding 2% (w/v) DSS in the drinking water for 7 consecutive days. At this concentration, DSS triggered a strong colonic inflammation accompanied by diarrhea and rectal bleeding. No mortality was observed. The administration of Lb CNRZ327 significantly attenuated diarrhea and rectal bleeding. In only one out of 4 independent experiments, weight loss was observed in the DSS group compared with the negative control group, and Lb CNRZ327 significantly reduced this weight loss. In other experiments we observed no weight loss after DSS exposure. Variability of this parameter after DSS treatment has been reported earlier [25]. At the histological level, the administration of Lb CNRZ327 strongly diminished the tissue damage induced by DSS, demonstrating its anti-inflammatory effects.

We previously reported that Lb CNRZ327 attenuates DSS-induced colitis in BALB/c mice [16]. In the present study, we observed an even stronger anti-colitis effect of Lb CNRZ327 in C56BL/6 mice. Although a direct comparison is precluded as we did not perform experiments using C56BL/6 and BALB/c mice in parallel in the same laboratory, the difference may be explained by the fact that these mice strains differ in their response to DSS due to their different genetic background [26].

The administration of Lb CNRZ327 tended to increase the level of gut secretory IgA (sIgA), although the difference with the DSS group was not statistically significant. Several other studies reported that the consumption of probiotics was associated with increased sIgA levels [23], [24] which could reinforce the gut barrier and limit the penetration of bacteria (commensal and pathogenic) into host tissues (reviewed in [27]). This is particularly relevant in the DSS model of colitis as DSS is directly toxic to gut epithelial cells and affects the integrity of the epithelial barrier [28], [29]. The mechanisms by which Lb CNRZ327 affects sIgA production remain to be elucidated.

Since cytokines are major mediators of inflammation and regulatory activity in the gut mucosa, we analyzed the ability of Lb CNRZ327 to modulate the production of cytokines in colonic tissue of the mice. TGF-β, IL-4, IL-6 and IL-12 were found to be significantly increased in the colonic tissue of DSS-treated mice when compared with the negative control. Lb CNRZ327 decreased the production of TGF-β, IL-6 and IL-12 to reach a level similar to that in the negative control. TGF-β1 is an abundant cytokine in the intestinal mucosa where it has immunosuppressive as well as pro-inflammatory properties. TGF-β1 can inhibit T cell proliferation, macrophage activity and secretion of several pro-inflammatory cytokines. Secretion of this cytokine can be triggered in regulatory T cells and epithelial gut cells as a protective mechanism to control tissue damage and overt inflammation. On the other hand, in combination with the pro-inflammatory cytokine IL-6, TGF-β can induce differentiation of Th17 cells, a T cell subset with a prominent pathogenic role in inflammatory bowel diseases [30]. Therefore, elevated levels of local TGF-β1 and IL-6 in colitis can be correlated with inflammatory events, fibrosis and structuring of the disease [31]. IL-12 is a pro-inflammatory cytokine that plays a pivotal role in the differentiation of Th1 cells and is upregulated in patients with IBD, especially those with CD [32], [33], while IL-4 is critical in generating CD4+ Th2 effector cells [34]. Interestingly, Mannon *et al.* [35] demonstrated that treatment with a monoclonal antibody against IL-12 induces remission in patients with active CD. Therefore, the reduced levels of TGF-β, IL-6 and IL-12 at the peak of colonic inflammation in mice treated with Lb CNRZ327 may be an indicator and explaining factor of the lower degree of inflammation in these mice.

The modulation of pro- and anti-inflammatory cytokines is an important mechanism underlying the effects of several probiotics although the actual cytokines affected may differ. While for *Lactobacillus casei* DN-114 001, for example, an increase in IL-10 seems to explain the observed anti-inflammatory effect [36], here a different mechanism appears to be involved. We observed clear anti-inflammatory effects, but no changes in colonic tissue IL-10 levels. Other studies have shown that several other lactic acid bacteria are, like *L. delbrueckii*, able to reduce the production of the pro-inflammatory cytokine IL-6 in the inflamed colon of mice [37]–[39].

In addition to cytokine levels in colonic tissue, DSS and Lb CNRZ327 affected cytokine levels in the spleen. TGF-β was increased in the spleen of DSS treated mice and, as observed in the colonic tissue, Lb CNRZ327 restored TGF-β levels to those found in the non-inflamed mice, demonstrating the ability of Lb CNRZ327 to modulate not only local but also systemic immune responses. No significant differences between mice treated with DSS or DSS and Lb CNRZ327 were found for the other splenic cytokines measured, except for IL-6 which was increased by ∼34% after administration of the bacterium, at a difference with the colon where Lb CNRZ327 caused a reduction of this cytokine. However, the latter difference should be regarded in the light of the DSS-mediated strong induction of IL-6 in the colon, which is nearly annulled by Lb CNRZ327, and the absence of IL-6 induction by DSS in the spleen. Lb CNRZ327 thus appears to correct the large change in IL-6 brought about by DSS treatment in the colon, while inducing a small raise of IL-6 in both the spleen and colon when compared to the PBS control.

Few studies have addressed the systemic immune responses triggered by orally administered bacteria. The consumption of *Lactobacillus fermentum* CECT5713 was reported to enhance the production of Th1 cytokines (especially IL-12) in the spleen of healthy mice while *Lactobacillus salivarius* induced IL-10 production [40]. Lavasani *et al.* [41] demonstrated that the oral administration of three strains of *lactobacilli* (*Lactobacillus paracasei* DSM 13434, *L. plantarum* DSM 15312 and *L. plantarum* DSM15323) prevented the signs of multiple sclerosis, a T cell mediated inflammatory autoimmune disease affecting the central nervous system, in mice, and that effect correlated with the attenuation of pro-inflammatory Th1 and Th17 cytokines followed by IL-10 induction in the mesenteric lymph nodes (MLN), spleen and blood.

In addition to the effects of Lb CNRZ327 on cytokine production, we evaluated two cellular parameters. CD4+ T cells expressing the Foxp3 transcription factor constitute a classical subset of regulatory T cells (Tregs) that play a crucial role in preventing the development and activity of inflammatory effector T cells. Natural CD4+Foxp3+ are generated in the thymus as key elements to regulate autoimmune reactivity in the body [42]. CD4+Foxp3+ T cells can also be generated in peripheral lymphoid sites and the gut associated lymphoid tissue is especially prone to induce the differentiation of these cells [42]. These cells were shown to be important players in the homeostasis of the gut mucosa [43], and individuals with IBD present altered frequencies of these cells [44], [45]. Our results show that DSS exposure leads to a decrease in the frequency of CD4+Foxp3+ T cells in the CLN and that Lb CNRZ327 is able to restore the frequency to a level similar to that in the negative control. Lb CNRZ327 did not affect the frequency of T cells expressing the activation marker CD69+, demonstrating that this strain is not able to activate CD4+ lymphocytes, thus confirming that Lb CNRZ327 does not induce inflammatory responses.

These results are reminiscent of those obtained with several probiotics, that attenuated colitis symptoms through the local expansion of different Tregs subtypes. The anti-colitis effect of *L. salivarius* 33 was correlated with the expansion of CD4+Foxp3+ T cells in the MLN [46] while VSL#3 increased the population of Tregs bearing surface TGF-beta in the form of latency-associated protein (LAP) (LAP+ T cells) in the lamina propria [14].

Here, in addition we observed a strong tendency of restoration of CD4+Foxp3+ regulatory T cells in the spleen of mice treated with DSS and Lb CNRZ327, as compared to mice treated with DSS alone, highlighting the systemic improvement of inflammation parameters by Lb CNRZ327. Expansion of regulatory T cells in the spleen had earlier been reported after treatment with *Lactobacillus plantarum*, but only in healthy mice (i.e. without induction of colitis) [47].

In conclusion, we confirmed the anti-inflammatory effect of the dairy *Lactobacillus delbrueckii* strain Lb CNRZ327 in DSS-induced colitis in mice, and showed some of the mechanisms underlying its probiotic effects, at the local and systemic level. For the cytokines and cellular parameters where Lb CNRZ327 has an effect, the bacteria tend to correct the changes brought about by the DSS treatment. To our knowledge, this is the first demonstration of systemic immune modulation effects exerted by a dairy *Lactobacillus* strain.
